# Systematic assessment of the quality and integrity of popular mental health smartphone apps using the American Psychiatric Association's app evaluation model

**DOI:** 10.3389/fdgth.2022.1003181

**Published:** 2022-09-29

**Authors:** Nikki S. Rickard, Perin Kurt, Tanya Meade

**Affiliations:** ^1^ School of Psychology, Western Sydney University, Sydney, NSW, Australia; ^2^ School of Psychological Sciences, Monash University, Melbourne, VIC, Australia

**Keywords:** mobile application (app), smartphone, self-help, depression, anxiety, mood, app evaluation, digital mental health

## Abstract

Mobile phones are playing an increasingly important role in supporting mental health, by providing confidential, accessible and scalable support for individuals who may not seek or have means of accessing professional help. There are concerns, however, that many apps claiming to support mental health do not meet professional, ethical or evidence-based standards. App store search algorithms favour popularity (reviews and downloads) and commercial factors (in-app purchases), with what appears to be low prioritisation of safety or effectiveness features. In this paper, the most visible 100 apps for “depression”, “anxiety” and/or “mood” on the Google Play and Apple App stores were selected for assessment using the American Psychiatric Association App Evaluation model. This model systematically assesses apps across five broad steps: accessibility, integrity, clinical and research evidence base, user engagement and interoperability. Assessment is hierarchical, with the most fundamental requirements of apps assessed first, with apps excluded at each step if they do not meet the criteria. The relationship between app quality and app store visibility was first analysed. App quality was also compared across four different app function types: mental health promotion or psychoeducation; monitoring or tracking; assessment or prevention; and intervention or treatment. Of the 92 apps assessed (after eight failed to meet inclusion criteria), half failed to meet the first criterion step of accessibility, and a further 20% of the remaining apps failed to meet the second criterion step of security and privacy. Only three of the 10 apps most visible on app stores met the criteria for research/clinical base and engagement/ease of use, and only one app fulfilled all five criterion steps of the evaluation model. Quality did not differ significantly across app function type. There was no significant correlation between app quality and app store visibility, which presents a potential risk to vulnerable consumers. The findings of this review highlight the need for greater accountability of app developers to meet, and report, at least minimum quality and integrity standards for their apps. Recommendations are also provided to assist users and clinicians to make informed choices in their selection of reputable and effective mental health apps.

## Introduction

Mental health issues have increased globally over the past decade (1), culminating most recently with a substantial surge due to natural disasters and the COVID-19 pandemic (2, 3). Public health systems are struggling to meet this increased need and are calling for greater integration of digital mental health solutions to achieve scalability (4). Digital mental health refers to the use of digital technologies (primarily the internet and smartphones) to facilitate the delivery of mental health information and care (5), potentially addressing the overwhelmed services, and the access and cost barriers to such services. Given it is estimated that the global penetration of smartphones is over 80% (6), digital mental health also provides a platform to offer a highly scalable and personalized means of delivering mental health support.

Digital mental health services can overcome some of the barriers for people seeking mental health support (7). Health support *via* the internet or smartphone is within reach of most people, including cohorts who might be less likely to access health services for geographical (remote or regional communities) or financial reasons (low socioeconomic status). Young people are increasingly using the internet on their mobile phones to seek help for mental health issues (8, 9), which is promising given this cohort is the most reluctant to seek professional help and have the highest incidence of mental health issues (10). Many young people find digital mental health service (particularly smartphone apps) engaging and intrinsically rewarding to use and empowering in self-managing their health. Seeking help through digital mental health services is also private and confidential, which is important given the perceived stigma associated with mental illness for some people.

Digital mental health services can also respond to challenges of limited access to face-to-face health services, for instance by providing real-time monitoring and risk detection outside of visits with a health practitioner, and by facilitating scalability of health promotion, prevention or early intervention services. Australia's Digital Mental Health Framework identifies how digital mental health services can be used as part of a stepped care model, responding to health promotion for the well population through to prevention and early intervention for at risk populations, as well as treatment for individuals with mild to moderate illness (11).

Research on mental health smartphone apps has demonstrated that, overall, there is sound evidence for potential effectiveness of mental health support through this delivery method. Largest effect sizes have been observed for apps which target depressive or anxiety symptoms (12–14) and for those that aim to improve quality of life and positive wellbeing (7, 12, 15). Apps are more likely to be effective if they include clinical guidance (13, 16), and these apps were found to be as effective as face-to-face support (13, 17). Stand-alone (that is without any human guidance) apps were, however, still superior to no support for depression, social anxiety, and stress levels, amongst other mental health issues (16).

Unfortunately, the app stores are highly volatile (18) and many of the apps with research evidence may no longer be publicly available (19). There are estimated to be over 20,000 mental health apps in app stores (20–22). However, very few of these have been subjected to any empirical or clinical scrutiny or have been published in scientific outlets. In a comprehensive search of mental health apps, Wang et al (23) identified that across 100 studies that have assessed mental health apps, only 14 apps had published evidence of their effectiveness. LeComte et al (12) estimate that less than 5% of all apps have any empirical support. Moreover, the apps that contain sound therapeutic foundation are not necessarily those that are being downloaded. For example, Wasil et al (24) found that while cognitive restructuring was present in 22% of the apps they reviewed, these apps reached just 2% of monthly users. In addition to clinical guidance, other factors likely to influence an app's effectiveness include the app's capacity to engage the user and a game-like feel, social interaction, self-monitoring, just-in-time reminders and personalised feedback (16, 25–28). Accessibility is, however, still a barrier for many users, with many apps including hidden or additional costs, privacy and security issues opaque to many users, and the digital divide still impacting a range of cohorts, including older individuals, indigenous peoples, socioeconomically disadvantaged and geographically remote individuals (11).

**Figure 1:**
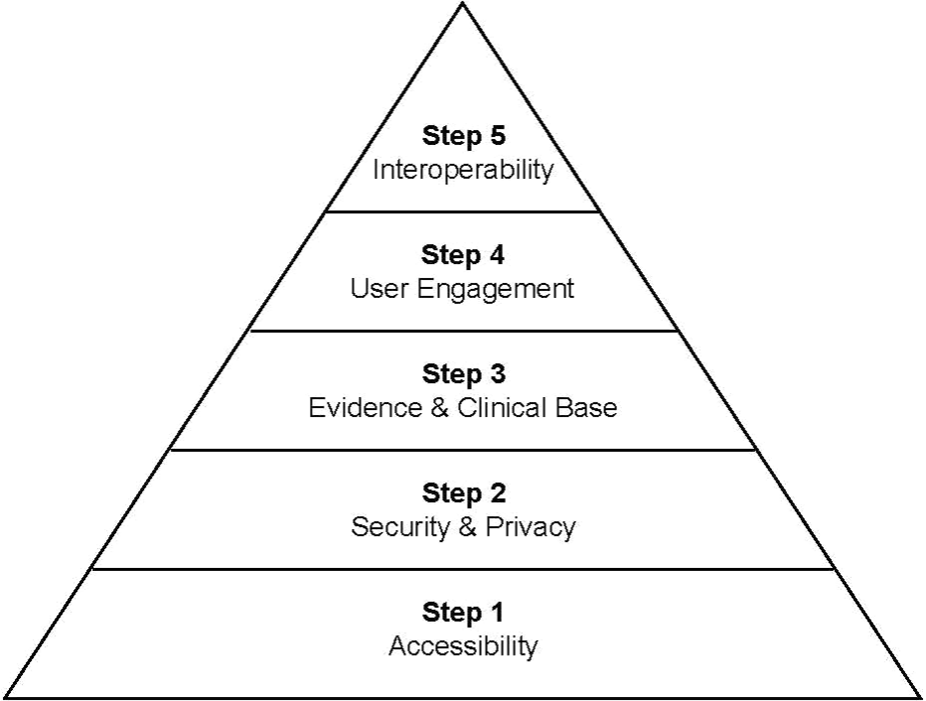
The APA evaluation pyramid (based on the pyramid model presented in Lagan et al, 2020) (38).

Consumers and clinicians therefore face a significant challenge in choosing accessible, credible, and safe apps to support mental health. In response, a number of assessment frameworks and tools have been developed to support objective evaluation of smartphone apps. For example, the Mobile App Rating Scale (MARS) is a widely used and easy to rate tool (29, 30), which focusses primarily on user engagement and design. App assessment hubs have also emerged, providing a repository of expert reviews of smartphone apps, including the Mind M-Health Index and Navigation Database (MIND), Open mHealth, Beacon, Mindtools.io, ORCHA and Psyberguide, as well as meta-repositories such as the European mHealth Hub. Expert reviews can provide an overview of an app in a format that is accessible to the wider public. However, given the release of regular app updates, such reviews may not provide an up-to-date evaluation (31). Common to most of these frameworks is a set of fundamental criteria on which apps should be assessed. These are well articulated in the comprehensive American Psychiatry Association (APA) Evaluation tool [(32); see Figure 1]. The APA evaluation tool is also the basis for the MIND website repository of publicly available and regularly updated smartphone apps (33).

Unfortunately, most consumers seeking mental health support on their smartphone are unlikely to visit app assessment hubs, or search for published research evidence prior to downloading an app. The primary means for selecting an app is through two app stores, Google Play Store (for Android devices) and the Apple App Store (for iOS devices), which accounted for 99% of the mobile operating system market share globally in May 2022 (https://gs.statcounter.com/os-market-share/mobile/worldwide. The app stores' search functions rank apps on the basis of an algorithm, which while not entirely transparent, appear to prioritise: (i) relevance to user: match between search word and app title, description and category, and the device they are using; (ii) user feedback: positive ratings, reviews, usage metrics (engagement, downloads); (iii) commercial features (in-app purchases, advertising, in-app events); and (iv) regular updates and technical performance of the app (Google: https://bit.ly/3QRfBki).

Features identified in assessment frameworks as important for mental health apps are not prioritised within app store search algorithms; evidence or clinical base is not included, nor are safety or interoperability features. There appears therefore to be a mismatch in the ranking of apps assessed by the professional mental health community as being useful and safe for mental health support, and those that will be visible to the consumer (or clinician) searching for mental health apps. One study has examined expert ratings (across three different app evaluation frameworks, ORCHA, MindTools.io and PsyberGuide) of the 25 most downloaded behavioural health apps (20). They found that quality ratings varied considerably across the evaluation frameworks, but that very few of the most popular apps were rated as high quality across all three frameworks.

The current study aimed to investigate the correspondence between quality of apps (as assessed by the APA Assessment tool) and the visibility of apps (as determined by popular search strategies on app stores). The scope of the study was limited to apps targeted at mood disorders, which are the most common forms of mental illness (34) and the most researched smartphone apps (35). Since full systematic reviews and meta-analyses exist elsewhere (12–14, 23, 27), this study applied a consumer lens to focus on apps most likely to be visible when performing common searches within the app stores, and therefore the most likely to be accessed by the general community.

## Materials and methods

The PRISMA guidelines for identifying and screening database searches (36) and The Protocol for App Store Systematic Reviews (PASSR) adaptation (21) were used to guide app selection in this study. Apps were identified across the two most popular app search stores using a set of commonly used mental health search health terms, and then ranked to yield a “top 100” of popular mental health apps. This set of apps was then subjected to the APA App Evaluation process, which involves exclusion of apps as assessment moves through a hierarchy of quality criteria.

### Inclusion and exclusion criteria

Apps were eligible for inclusion if they were available on either the Apple App or the Google Play Store and if they met the inclusion criteria outlined below:

1. Apps are available in the English language.
2. Apps are included regardless of whether they are paid, contain in-app purchases or are free, and regardless of when they were launched or updated.
3. The apps' primary aim is to support wellbeing promotion, or prevention or treatment of depression or anxiety, and this must be discernible from the app title or description in the app store search page.
4. The apps must be designed as a standalone service, without the need for human support (e.g., telehealth counsellors or web-based service) or additional devices (e.g., wearable heart-rate monitor).

Apps were excluded if they were designed for exclusive use by universities (for research purposes), health professionals (rather than individual users) or specific health care services and clinics (requiring a unique login for that service).

### Search strategy and ranking of app store visibility

The Apple App and Google Play stores were searched in December 2021. A two-stage Search strategy was used to ensure the most frequently viewed apps were identified (37).

Stage 1 consisted of a search in each store for the individual terms: “depression”, “anxiety” or “mood”. (The first search output in the Apple App Store appeared to be sponsor-generated, so was not included unless it appeared again in subsequent search outputs.) The lists from the two stores were then combined and ranking as assigned by each app stores recorded. For example, the most visible (first appearing app) in each store was given a rank of “1”, and the second appearing app in each store was each given a rank of “2”. Duplicates across search terms and app stores were removed.

Stage 2 searched in each store for the string of terms “depression anxiety mood” (Boolean search terms such as “OR” do not appear to be recognised on the Apple App Store so the combined search involved all three words separated by a space). Again, the app store lists were combined, maintaining equal ranks across stores, with duplicates across stores removed.

The two lists were then combined, and the top 100 most visible apps selected for assessment. As ranking occurred across two app stores, and for four search terms, up to eight different apps could be assigned an equivalent rank.

### APA assessment criteria and coding procedure

The full APA tool (32) includes 105 criteria items assessing an app's accessibility, privacy and security, clinical foundation, engagement, and interoperability (38). The framework encourages beginning assessment with the most fundamental step of the criteria (accessibility), and then working up through five steps. The shorter APA tool (see https://www.psychiatry.org/psychiatrists/practice/mental-health-apps/the-app-evaluation-model) includes eight items, which enables initial screening, for instance if a large volume of apps needs to be reduced to a shortlist. For this review, the eight items forming the shorter screening tool were selected (see Table 1). An additional six items from the full tool (but missing in the screening tool) were also included to ensure each of the key constructs covered in reviews on apps were assessed in this study. These included items on: transparent detail on storage of data, a disclaimer that the app is not a replacement for professional services, credibility of the app developer, published research base, engagement features of the app and whether the app can be tailored for individual users. Items were grouped according to the five steps of the APA assessment model (accessibility, privacy and security, evidence and clinical base, engagement and ease of use, interoperability), with each step comprised of 1–4 items. Each app received a possible rating from 0 to a maximum of 14.

**Table 1:** Selected items from the APA's screening assessment tool used in the systematic assessment of apps in this review (consisting of eight items from the Screening tool plus an additional six items to ensure each criterion step well represented).

Step	Criteria	Rating Guide
Step 1: Accessibility *Can we access adequate information about the app?*	1. On which platforms/operating systems does the app work?^a^	App was available on both Apple iStore and Google Play store (two dominant app stores). There was no requirement for it to also be available for desktop browser.
	2. Has the app been updated in the last 180 days?^a^	Search was performed at start of January 2021, so apps had to have been updated since July 2021 (6 months∼180 days).
Step 2: Security and Privacy *Does the app demonstrate sufficient data privacy and security? Is the app ethical?*	3. Is there a transparent privacy policy that is clear and accessible before use?^a^	A Privacy policy that was transparent - clear and accessible—was accessible prior to downloading app (ie. on App store); in English and comprehensible.
	4. Does the app declare where the data is stored?^b^	Privacy policy stated how any personal information would be collected and used.
	5. Does the app collect, use, and/or transmit sensitive data? If yes, does it claim to do so securely?^a^	Privacy policy stated where the information being collected would be stored and if that storage was secure.
	6. Does the app include qualification that the app is not a replacement for professional services?^b^	Statement was clear and prominent enough on app store description or first page of website for app to be noticed.
Step 3: Evidence and Clinical Base *Does the app have sufficient clinical foundation? Is the app based on research evidence?*	7. Is the source of the app credible?^b^	Source of the app was cited on App store or website for app; association with university, medical centre, government affiliation etc. (rather than ambiguous, commercial or just an individual psychologist/psychiatrist) was regarded as evidence of credibility.
	8. Is there published research?^b^	Relevant sources or references supporting the app needed to be easily found *via* a search on either the app's website (e.g., link to Research) or on Google Scholar. “Grey literature” (conference abstracts, press blogs, company reports or papers) were not deemed relevant/credible.
	9. Does the app appear to do what it claims to do?^a,c^	Downloading the app and using it demonstrated that it had face validity—it did what it claimed it did.
	10. Is there evidence of specific benefit from credible sources?^a,c^	Research evidence, academic institution or user feedback demonstrated a specific benefit of app use.
Step 4: User Engagement *Is the app user-friendly?*	11. Does the app seem easy to use?^a^	Once downloaded, app was easy enough to work out how to start using it.
	12. How engaging is the app?^b^	App was subjectively enjoyable and engaging; typically this meant it utilised multi-media rather than just text, and was interactive (e.g., gamified), and attractive.
	13. Is it customisable/personalised?^b^	App allowed individual users to adjust settings for their own personalised use, or the app demonstrated that it adapted to different users, providing tailored feedback or other adaptive use pathways.
Step 5: Interoperability *Can the data be shared*	14. Can data be easily shared and interpreted in a way that is consistent with the stated purpose of the app?^a^	App included options to export or share their data with a third party (e.g., their clinician).

^a^ Shorter APA screening items.

^b^ Additional items.

^c^ Items that were reworded.

A hierarchical process was used such that only apps meeting all items within an evaluation step were then assessed at the next step. While this process may not be applicable for all evaluation purposes (see the MIND database: https://mindapps.org/ for additional recommendations), it prioritises user safety by prioritizing non-maleficence and equity (39). Each app was also classified according to its primary mental health support function; that is, mental health promotion or psychoeducation; monitoring or tracking; assessment or prevention; and intervention or treatment (including peer support).

Two independent raters assessed all apps on each of the criteria in Table 1 of the APA Evaluation tool. Two authors (NR and PK) first discussed each of the items together to identify what would constitute meeting each criterion and evaluation step. Research Assistants (one of which was the author PK, and the other an independent research assistant) then rated the apps. At each step, two of the authors (NR and PK) met to confirm which apps had met all the criteria for that step and therefore proceeded to be evaluated at the subsequent step. Ratings for any apps on which the raters disagreed were also discussed at this point, and agreement was reached. Inter-rater agreement ranged from 71% to 100% across the five criteria, with the lowest agreement being for Step 4 (reflecting the subjective elements of the “Engagement” criteria).

### Analysis of app visibility and quality

Quality ratings were compared across app category types using a one-way ANOVA (two-tailed, *α* = .05). Spearman's correlations (*α* = .05) were performed to assess the association between the app popularity and the apps quality rating (operationalised by score out of 14).

## Results

### Search results

**Figure 2:**
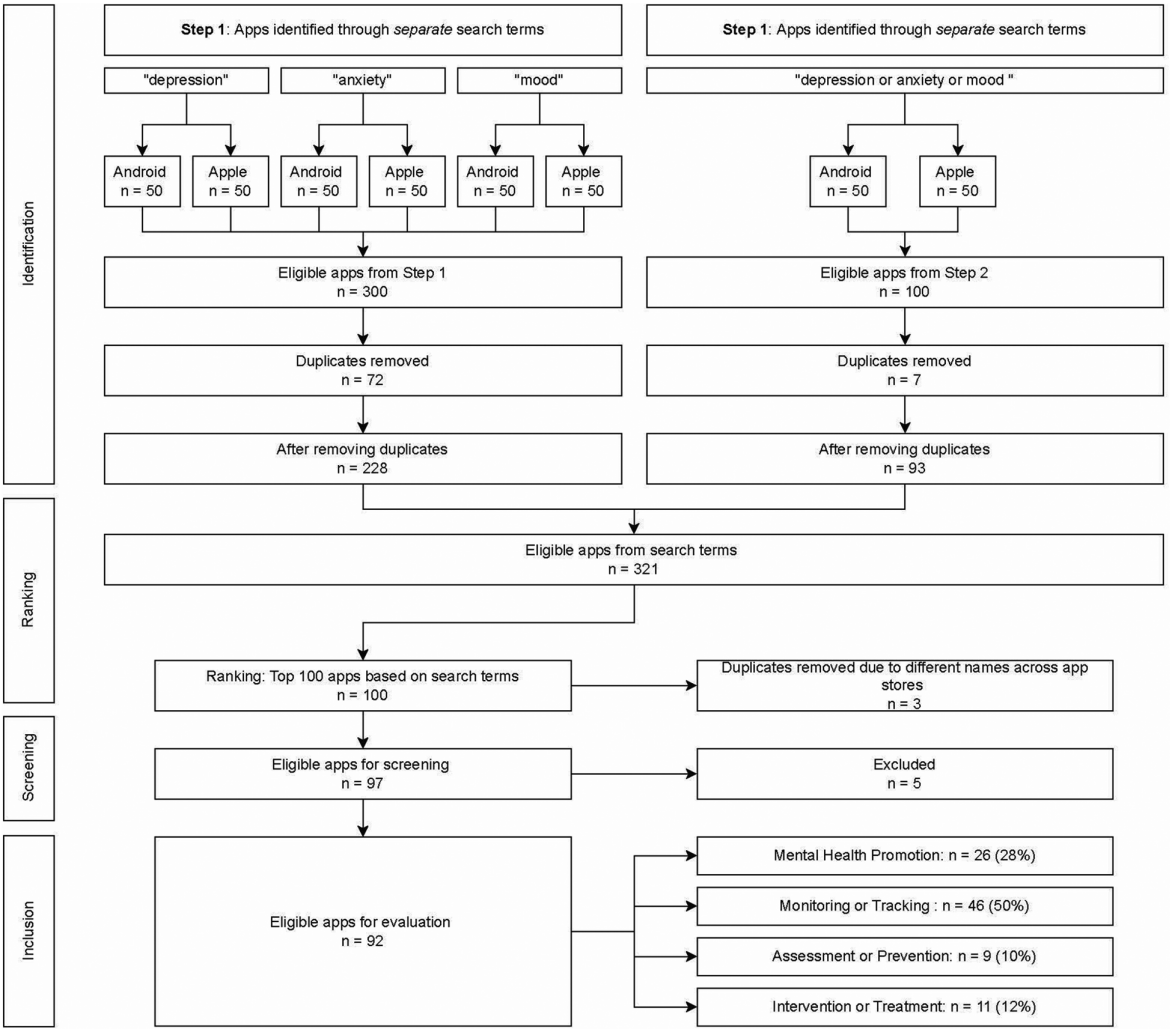
Search flow chart.

The initial search collated the first 50 apps within each search term, within each app store, generating a total of 400 “visible” apps. Once duplicates were removed, the top 100 “most visible” apps were selected from the remaining 321 apps. Screening for exclusion criteria removed a further eight apps, leaving a total of 92 apps for evaluation (see Figure 2). As ranking occurred across two app stores and in response to four search terms, multiple apps were assigned the same rank. This yielded a total of 40 differentiated ranks, with all apps being assigned a rank from 1 (most visible) to 40 (least visible).

### Characteristics of included apps

The 92 apps included in the evaluation are listed in Appendix A. The apps were categorised by their primary function. Apps delivering health information or incorporating gamification techniques were classified as “mental health promotion/psychoeducation” apps. Self-monitoring apps were classified as “monitoring/tracking” apps. Apps involving therapeutic exercises were classified as “intervention/treatment” apps, and apps which assisted users to screen or test their mental health or wellbeing were classified as “assessment/prevention” apps.

#### Frequency of apps meeting assessment criteria

**Figure 3:**
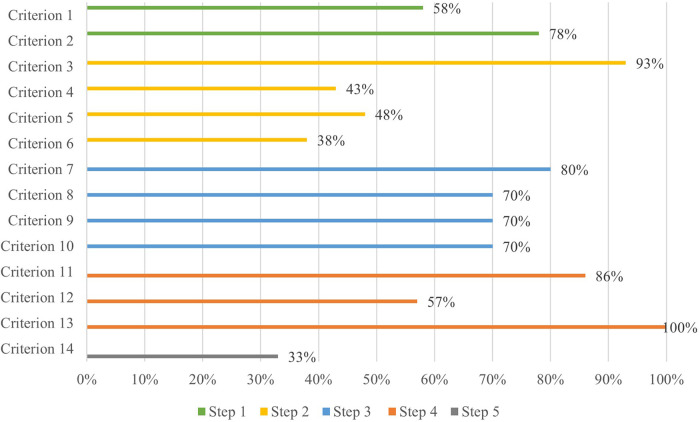
Assessment of apps using the APA framework—percentage of apps within each of the 5 steps meeting each criterion item. (Note. Step 1 = Accessibility, Step 2 = Security / Privacy, Step 3 = Evidence / Clinical Base, Step 4 = User Engagement, Step 5 = Interoperability).

**Figure 4:**
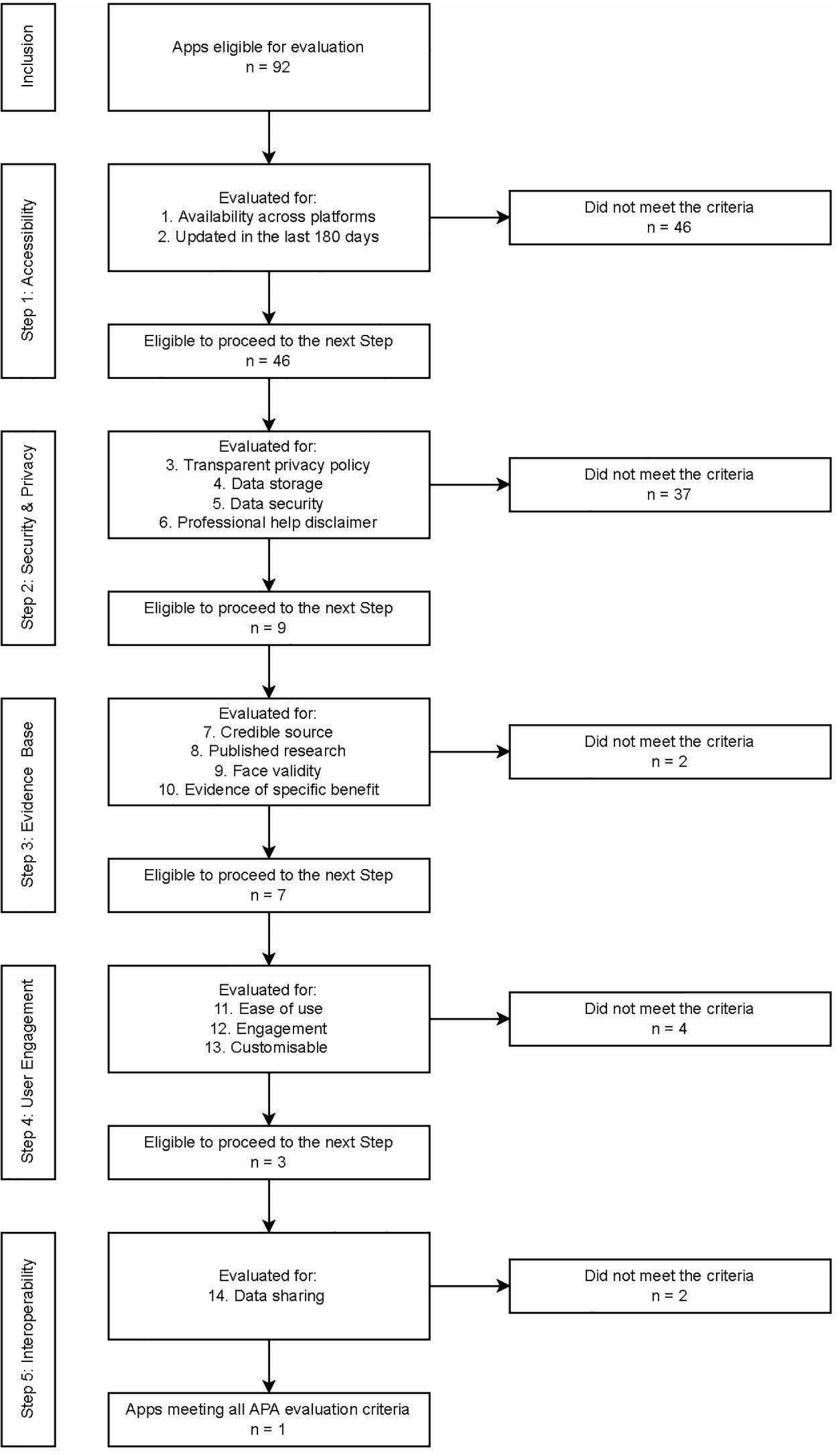
Progression of 92 apps across each evaluation step.

The majority of apps assessed achieved a quality score of less than seven, indicating that they were excluded from further assessment by Step 2 of the APA framework (see Figure 3). When broken down by proportion of apps meeting each of the criteria within each step, the majority of apps had been updated recently (78%) and over half were available on both major app platforms (58%). However, only 46 apps (50%) met both criteria in Step 1 (see Figure 4). The majority of those 46 apps (93%) then met the Step 2 (security) criterion of having a transparent privacy policy, but less than half the apps met the remaining three criteria (transparent storage information, secure collection and use of data, and disclaimer that app was not a replacement for professional services). Subsequently only nine apps from Step 1 met all four criteria items of Step 2.

**Figure 5:**
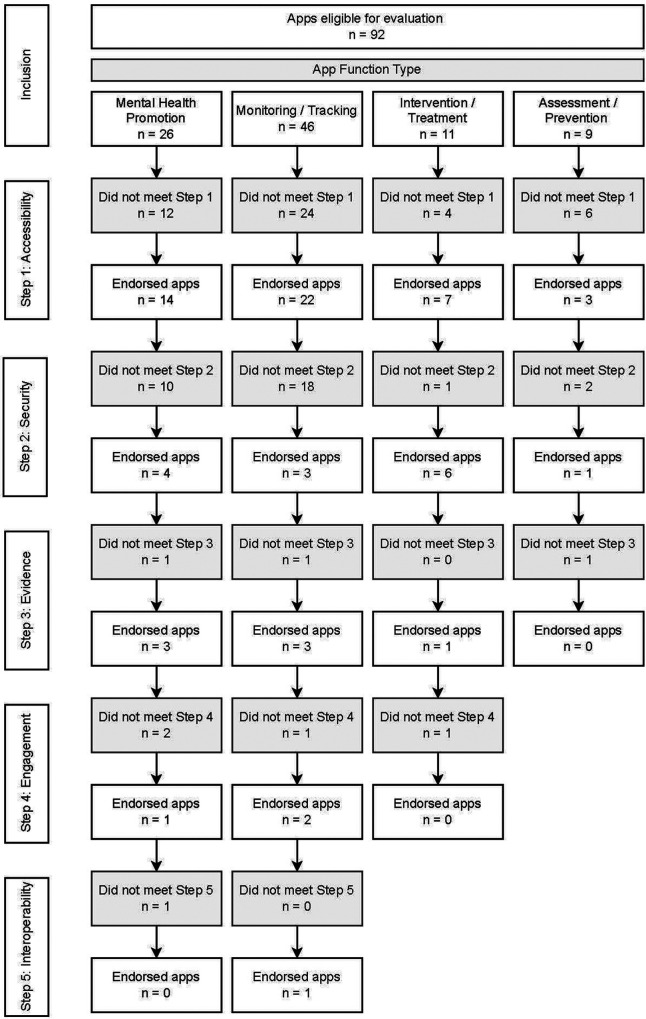
Quality assessment of 92 apps grouped by their function type.

Step 3 (evidence base) further excluded another two apps based on insufficient credibility in the development team or face validity, and lack of research performed to evaluate the impact of the apps. The majority of the seven remaining apps also met two of the criteria in Step 4 (user engagement) relating to ease of use and personalisation to the user, but a further three were excluded for not meeting the criterion of being engaging. By Step 4, only three apps (MindDoc, Wysa and GG OCD Anxiety and Depression) (3% of the original pool of 92) met the criteria and proceeded to Step 5. Two of the final three apps evaluated did not enable easy sharing of data with an external party, such as a clinician, leaving only one app (MindDoc) fulfilling all five steps of the evaluation (see Table 2 for a brief description of the app). This represents only 1% of apps from the original pool meeting the criteria of all five steps of the evaluation (see Figures 4, 5).

**Table 2:** Qualitative description of app which met all five APA evaluation tool criteria.

**Spotlight on a Quality Mental Health App: MindDoc** MindDoc (https://minddoc.com/us/en) was ranked 1 indicating it was highly visible on both the Apple App and Google Play stores, and that it also appeared in response to multiple search terms (“depression”, “anxiety”, “mood” or a combination of these terms). MindDoc is updated regularly and accessible to both iOS and Android users. The purposes for which personal information is collected, stored, and disclosed are clearly described in the privacy policy. MindDoc disclaims that the app is not a substitute for professional help. The app was developed by psychologists, has published research evidence designed to explore its efficacy, and guides users to recognise and understand the signs and symptoms of mental illness through evidence-based courses and exercises. The app is easy to navigate and provides personalised insights. MindDoc also allows users to export and share their data with their mental health provider.

#### Frequency of apps meeting assessment criteria by app function type

**Figure 6:**
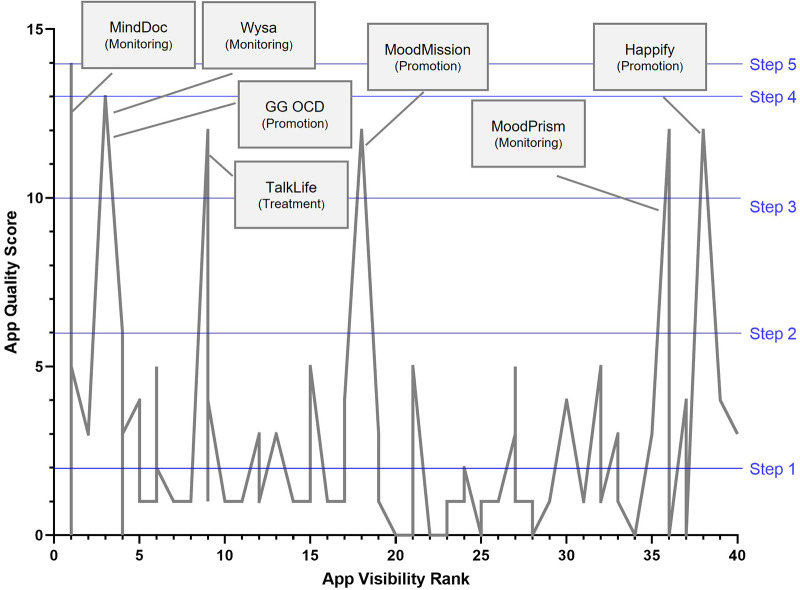
Association between apps visibility ranking (x-Axis, with rank of 1 being the most visible app on app stores) and app's quality score (out of maximum 14). Assessment criterion steps marked in blue lines, and highest quality apps flagged with callout descriptions. Note. That several apps can be ranked equally (as a result of multiple search terms and two app stores used), so each rank can demonstrate a range of quality scores).

Apps were categorised into four function types: mental health promotion/psychoeducation (*n* = 26), monitoring/tracking (*n* = 46), intervention/treatment (*n* = 11) and assessment/prevention (*n* = 9). Figure 6 shows the relative progression through the five assessment steps for each app category type.

Across the four types of apps the only app that met all five steps was in the monitoring/tracking category, which was also the largest group of apps (46/92), followed by the second largest group (26/92)—the mental health promotion/psychoeducation apps—from which two apps met all four steps. The remaining two categories (intervention and prevention) were very small (11/92, 9/92 respectively), with one app from each category making it through to Step 3. This analysis demonstrates that the vast majority of apps fail to meet the fundamental criteria of basic accessibility (50% of 92 apps at Step 1), and security and privacy transparency (33% of 46 apps at Step 2).

### App quality ratings

The app quality rating was calculated by summing the total number of criteria met out of a possible total of 14. The mean across all apps was only 3.02 (*SD *= 3.27, *n* = 92), reflecting a failure of most of the highly visible depression, anxiety and mood apps appearing in the apps stores to advance beyond the first two steps of the APA evaluation pyramid.

#### Correlation between app visibility and quality

The relationship between app visibility on app stores (ranked 1–40, where 1 is the most visible or popular apps and with subsequent ranking being indicative of diminishing visibility) and quality as assessed by the APA app Evaluation framework (total score out of 14) is illustrated in Figure 6. Overall, no clear association between app visibility rank and app quality appears to be present. A Spearman's correlation between app store visibility ranking and app quality (rating out of 14) was not significant, *r_s_* (*n *= 92) = −.17, *p* = .109.

Seven apps (MindDoc, Wysa, GG OCD Anxiety and Depression, TalkLife, MoodPrism, Happify and MoodMission) emerged from the assessment as of substantially higher quality than the rest, meeting all criteria for the at least the first three steps in the APA evaluation framework. The apps were distributed across the entire visibility rank range (with MindDoc highly visible on App store searches, while Happify was much less visible), and represented three of the four category types (monitoring, promotion and treatment). Three of the seven highest quality apps were also ranked amongst the highest in visibility on the app stores. Conversely, a number of low-quality ranking apps were also highly visible, with one app ranking 1 on visibility that received a quality score of 0, demonstrating it had not even met the fundamental criteria of accessibility (due it being accessible on only one of the major app stores).

#### App quality by app function type

Across the four app function types, the average quality score for mental health promotion apps was highest (mean quality score = 3.50, *SD* = 3.76, *n *= 26), followed by intervention/treatment apps (mean quality score = 3.27, *SD* = 3.41, *n *= 11), monitoring/tracking apps (mean quality score = 2.93, *SD* = 1.92, *n *= 46), and with assessment/prevention apps yielding the lowest average quality score (mean quality score = 1.78, *SD* = 1.92, *n *= 9). A one-way ANOVA (homogeneity of variances assumption met), however, demonstrated no significant differences between quality scores across these categories in this sample, *F*(3,88) = 0.64, *p* *=* *.589*.

## Discussion

The aim of this study was to examine the correspondence between app quality as assessed by the APA assessment tool and the visibility of apps as determined by popular search strategies on app stores. The scope of the included apps was focused on apps that targeted depression, anxiety, and mood as these are the most common mental health conditions. Of the 100 apps that were selected based on visibility, 92 were progressed to quality assessment. Of those 92, only 14 (15%) met the first two criteria steps, being *accessibility*, and *security and privacy* criteria. While a common concern regarding mental health apps is cited to be the absence of research evidence, the analyses reported in this paper show that far more basic criteria are not being met by many apps being downloaded by consumers.

Subsequently, half (seven out of 14) met the next quality criteria, evidence (credibility, research, validity, benefits), which is less than 1% of the original sample of apps. This is consistent with the findings across other studies (12, 23). Of those seven apps, function-wise, three were promotion, three were monitoring and one was treatment focused. Only three apps progressed to Step 4, *engagement*, and only one app, MindDoc, met the last Step 5, *interoperability*. The relationship between the apps' quality scores and apps' visibility was not significant, as can be seen in the Figure 6, and notably in the positioning of the top seven apps, as based on APA's assessment, across the visibility range. Similar findings were reported in other studies (24). Visibility, which may be determined by several indicators, including popularity, is therefore not indicative of quality of apps.

Given how very few of the reviewed apps met the five steps of criteria assessment, it may be that the apps are poorly designed, and/or that the APA assessment tool is too critical for most apps to meet. However, most apps failed to meet the *accessibility* and *security and privacy* criteria, which are basic app development features and therefore at the entry level of the assessment hierarchy. *Accessibility* which includes platform access and currency of the app, is a baseline requirement as should *security and privacy* be. However, those criteria may not be of equal importance to the user or to the health service provider who may recommend an app based on some other features, such as its purpose or perceived effectiveness.

In the current study, it appears that if apps can meet those two basic quality criteria (steps 1 and 2) they are more likely to demonstrate *evidence* (Step 3) as 80% did. While based on a small sample of nine apps (less than 10% of the initial sample of apps) this is a positive finding. The next assessment step (Step 4), *engagement*, led to further reduction with more than 50% of those remaining apps failing to meet it. Users' engagement with an app and its range of features, is critical to the potential effectiveness of the app (16, 26–28). It is also a standard feature of app development irrespective of the topic or the purpose of the app. However, there are individual differences in how an app may be used even if it has a high range of engaging features. Those individual differences in which engagement features may or may not be valued is evident in users' reviews and may or may not align with the quality assessment tools.

Similarly, the final and fifth step, *interoperability* while at the top of the assessment hierarchy may have varied context application with features, such as data sharing, not being applicable to some apps (i.e., promotion or prevention vs. treatment focused) or not being critical to some users or some health services providers. Those individual and contextual differences are important considerations in selection and recommendation of apps. Future research could explore who different users view quality assessment and to what degree those views are aligned with the assessment tools such as APA's.

The APA five steps criteria assessment tool is comprehensive and informative, but there is a level of complexity involved in identifying the range of apps and then assessing them across those quality criteria. Both of those tasks would be outside of the informed decision-making scope of users or clinicians. Both groups, however, could, be assisted by provision of a practical “short list” guide to essential checks that could include, for example:

1. “About this app”: Update date—Was it within last 6 months? [Accessibility].
2. “About this app”: Disclaimer or description—Is the app equipped to respond to potential safety concerns—for example, does it call out that this app is not a replacement for professional services? [Privacy and Security].
3. “See details”: Privacy Policy—Can you find anything about how your personal information is collected, used and shared? [Privacy and Security].
4. “Ratings and Reviews”—Does the app have at least 4 stars? Read a few reviews—Does it sound like it's easy/enjoyable to use? [Engagement].
5. “Google Scholar” webpage (https://scholar.google.com.au or a similar research publication database)—search for the app—Are there any high-quality published articles about the app? [Evidence].

Potential users of such apps could then make an informed choice as for some, privacy and security may not be a critical limitation and an app that has a high engagement feature can be critical to usage of the app and its potential to engage an individual in self-care, health knowledge and pathway to health services access, despite not meeting other quality criteria. The M-Health Index and Navigation Database (MIND; https://mindapps.org/) resource is a customisable and searchable database of app features which have been evaluated using the APA Evaluation framework (38, 40). It allows the user to prioritise which features are of most value to them, allowing informed choice. However, a simpler tool which could be embedded into app stores or made more easily available for consumers may be more likely to be utilised by everyday users. In addition, clients are more likely to adopt digital mental health tools when recommended by a clinician (41), so raising awareness of tools like the MIND resources in clinical training may help support clinical integration.

Apps can play a significant role in health prevention, promotion and treatment in the scope and reach they offer (42). Currently, most do not meet basic or comprehensive quality and integrity checks. This aligns with clinicians' reluctance about using mental health apps in their practice, with many reporting concerns about data security and clinical safety (22, 43). In the context of apps being used to aid health service provision from promotion to prevention to treatment, health service providers need to be aware of the core indicators of quality and integrity of such apps and provide informed recommendations that are specific to their patients and clients' health support needs (44–46). Development of mental health apps would benefit from an application of APA quality assessment to improve their capacity and potential place in the public health support system. Future research could examine to what degree APA's quality check is implementable in the app designing and development sphere which is influenced by time, cost, scalability and sustainability challenges.

This study focused on assessment of apps based on their visibility. This is both a strength and a limitation. Visibility is an indicator of popularity and access but also of algorithms that may include features that are neither indicative of quality nor of preferred usability. Other studies have utilised different methods, including popularity as measured by the number of downloads and users' review (47–49). Users' reviews may be particularly informative as individual perception of the purpose of a particular app and how they engage with such an app can be based on a different set of quality criteria to that of the app developers or the health service providers. In this context, publicly available training advice could be a valuable resource to assist raters tailor their assessment to the purpose of the app.

In summary, the APA assessment model provides a structured approach to determining quality and integrity of apps. In this study, the visibility of apps was not aligned with the quality of those apps, however a small number of apps met most of the APA assessment criteria. Further research may investigate the relationship between the quality of apps based on the APA assessment and the effectiveness of those apps.

## Data Availability

The raw data supporting the conclusions of this article will be made available by the authors, without undue reservation.
