# Supplementary material for: Systematic assessment of the quality and integrity of popular mental health smartphone apps using the American Psychiatric Association's app evaluation model
Source: Front Digit Health. 2022 Sep 29;4:1003181. doi: 10.3389/fdgth.2022.1003181 (PMC9561256; doi:10.3389/fdgth.2022.1003181)
Supplement: Supplementary file 1 [file TableS1.docx]

# Appendix A: Characteristics of Assessed Apps. (P: Promotion, M: Monitoring, A: Assessment/Prevention; T: Intervention/Treatment; App: Apple App Store, G: Google Play Store).

| **App Store Visibility Rank** | **App Name** | **App Publisher** | **App Function Type** | **App Platform Availability** | **App Score Out of 14** |
| --- | --- | --- | --- | --- | --- |
| 1 | Inner Hour: Self-Care Therapy | InnerHour | P | A & G | 7 |
| 1 | MoodSpace | Chachi Productions | P | G | 1 |
| 1 | Depression Test | Baris Sarer | A | A | 2 |
| 1 | MindDoc: Your Companion | MindDoc Health | M | A & G | 14 |
| 1 | Dare: Anxiety & Panic Relief (also called Relieve Your Anxiety with DARE) | BMD Publishing | M | A & G | 4 |
| 1 | Daylio Journal (also called Daylio Journal – Mood Tracker) | Habitics | M | A & G | 5 |
| 2 | Sanvello: Anxiety & Depression | Sanvello Health Inc. | P | A & G | 3 |
| 3 | Wysa: Anxiety, Therapy Chatbot (also called Wysa: Mental Health Support) | Touchkin | M | A & G | 13 |
| 3 | GG OCD – Anxiety & Depression | Ggtude Ltd | P | A & G | 13 |
| 4 | Depression Test^+^ | Inquiry Health LLC | A | A & G | 6 |
| 4 | Anxiety Release based on EMDR | Trauma and Pain Management Services Pty Ltd | T | A & G | 3 |
| 4 | DailyBean - Simplest Journal (also called DailyBean – The Simplest Journal to Record a Day) | BlueSignum Corp. | M | A & G | 5 |
| 4 | Control and Monitor: Anxiety, Mood and Self-Esteem | TwoCats App | M | G | 1 |
| 4 | MoodLinks - Anxiety & Depression | Recovery Records | M | A & G | 4 |
| 5 | uMore (also called uMore – Mental Health Tracker) | uMore | M | A & G | 4 |
| 5 | Mood Balance: Self Care Tracker | Harmonybit Ltd | M | A | 2 |
| 6 | Anxiety Tracker – Stress and Anxiety Log | Appstronaut Studios | M | G | 2 |
| 6 | Breathwrk: Breathing Exercises | Breathwrk Inc. | P | A & G | 5 |
| 6 | The Mood Meter | The Emotionally Intelligent Schools LLC | P | A & G | 2 |
| 6 | Breeze: Moodboard, Journaling (also called Breeze: Mood Tracker) | Basenji Apps | M | A & G | 2 |
| 7 | Bloom CBT Therapy & Self-Care | Meemo Media Inc. | T | A | 2 |
| 8 | Moodnotes – Mood Tracker | ThrivePort, LLC | M | A | 2 |
| 9 | TalkLife | TalkLife Ltd | T | A & G | 12 |
| 9 | Headspace: Meditate & Relax (also called Headspace: Mindful Meditation) | Headspace Inc. | P | A & G | 5 |
| 9 | EMMO (also called EMMO – Mood Diary) | EMMO Corp. | M | A & G | 5 |
| 9 | CBT Guide to Depression | Excel at Life | P | G | 4 |
| 9 | Reflectio: AI Mood Tracker | Gototop LTD | M | A & G | 4 |
| 10 | Mood Tracker for Mental health | Reflexio | M | G | 2 |
| 10 | UpLift – Depression & Anxiety | UpLift Health Inc. | P | A | 2 |
| 11 | Mind Journal: Diary, Mood Tracker & Gratitude | Bazimo | M | G | 2 |
| 12 | Anxiety Test | Inquiry Health LLC | A | A & G | 3 |
| 12 | Moody: Mood Tracker & Journal | Media Studios | M | G | 4 |
| 13 | Woebot: Your Self-Care Expert in CBT & Mindfulness | Woebot Health | P | A & G | 4 |
| 14 | Finch: Self Care Widget Pet | Finch Care Inc | P | G | 3 |
| 14 | Worry Watch: Anxiety & Mood | Akilan Rajendran | M | A | 2 |
| 15 | MoodTools - Depression Aid | Inquiry Health LLC | M | A & G | 1 |
| 15 | CBT Thought Diary | Inquiry Health LLC | M | A & G | 5 |
| 15 | MindShift CBT - Anxiety Relief | Anxiety Canada Association | T | A & G | 6 |
| 15 | Worrydolls | Peter Wieben | M | A & G | 3 |
| 15 | eMoods Bipolar Mood Tracker | Yottaram LLC | M | A & G | 5 |
| 15 | Reflectly - Diary, Gratitude Journal & Mood Tracker (also called Reflectly – Journal & AI Diary) | Reflectly ApS | M | A & G | 5 |
| 16 | Dealing with Depression | Fatbelly | P | G | 5 |
| 16 | Anxiety Solution & Relief | PSYT | P | A | 2 |
| 17 | Mental Health Tests | Mind Diagnostics | A | A & G | 4 |
| 17 | BetterHelp – Therapy | BetterHelp | T | A & G | 5 |
| 18 | MoodMission (also called MoodMission – Cope with Stress, Moods & Anxiety) | MoodMission | P | A & G | 12 |
| 19 | Unwinding Anxiety | MindSciences Inc | P | A & G | 3 |
| 19 | Mood Patterns – Mood Tracker & Diary with Privacy | Mood Patterns | M | G | 2 |
| 20 | Depression Test | Japps Medical | A | G | 2 |
| 20 | MoodWell: Moody Tracker Diary | Pranoy Chowdhury | M | A | 1 |
| 21 | Simple Depression Test | Yu Zhang | A | A | 2 |
| 21 | Anxiety Relief by Mind Ease | Mind Ease Labs Ltd | A | A & G | 3 |
| 21 | AntiStress & Relaxing Games | Content Arcade (UK) Ltd | P | A | 5 |
| 21 | Breeze: mental health | Basenji Apps | M | A & G | 3 |
| 21 | Youper Therapy | Youper, Inc | T | A & G | 3 |
| 21 | Symptom & Mood Tracker | Bearable | M | A & G | 5 |
| 22 | MoodKit | ThrivePort, LLC | P | A | 1 |
| 23 | Anxiety Relief Hypnosis - Stress, Panic Attacks | Surf City Apps | T | G | 1 |
| 23 | Zen: Guided Meditation & Sleep | MoveNext, Ltd | T | A | 2 |
| 24 | Bipolar Test | Inquiry Health LLC | A | A & G | 3 |
| 24 | Stop Panic & Anxiety | Excel at Life | P | G | 6 |
| 24 | Moodflow: Mood Tracker | Monecke Labs | M | A & G | 2 |
| 25 | Mood Log | AR Productions Inc. | M | G | 1 |
| 25 | Moodistory – Mood Tracker | Christoph Matzka | M | A | 5 |
| 26 | Tappy: Self Care Fidgeter | Cameron Nazemi | A | A | 2 |
| 27 | Tochi - Mood Tracker, Journal | The Lazy Hippo Development | M | A & G | 3 |
| 27 | 7 Cups - Anxiety & Stress Chat (also called 7 Cups: Online Therapy & Chat) | 7 Cups of Tea | T | A & G | 5 |
| 27 | Depression Manager | @Point of Care | M | A | 7 |
| 27 | Moodz Hub – Mood Tracker Diary | Tycoon Tycoon Company Limited | M | G | 5 |
| 28 | Anxiety Tracker – Stress Relief | Technogic App Lab | M | G | 5 |
| 28 | Mood Mint – Boost Your Mood | Jason Pegg | T | A | 1 |
| 29 | Moodtrack Social Diary | Matthew Windwer | M | A & G | 1 |
| 30 | Chiku – Journal / Diary & Mood Tracker | chiku.app | M | A & G | 4 |
| 30 | VOS – Wellbeing & Mood Journal (also called VOS – Your Wellbeing Plan) | VOS.health | M | A & G | 4 |
| 31 | Ripple: Mood & Health Tracker | Bloomy Lab | M | A | 2 |
| 32 | MyPossibleSelf: Mental Health | My Possible Self Ltd | P | A & G | 5 |
| 32 | Self-Manage Depression (also called Self-Manage Depression: Daily Exercise) | GGDE | P | A & G | 1 |
| 32 | Flow – Depression Treatment | Flow Neuroscience | P | A & G | 6 |
| 32 | Sayana: Daily Self-Care Guide | Sayana Inc. | M | A | 2 |
| 33 | Rootd - Panic Attack Relief (also called Rootd – Anxiety & Panic Relief) | Simply Rooted Media | P | A & G | 3 |
| 33 | Pixels: Mental Health and Mood | Teo Vogel | M | A & G | 3 |
| 33 | vui - mood track | Diem Phuc An Nguyen | M | A | 5 |
| 34 | PsychApp – Depression, Anxiety, Panic Attack | ArtSmart Software | M | G | 2 |
| 35 | DBT Coach | Resiliens, Inc | T | A & G | 3 |
| 36 | MoodPrism | MoodPrism | M | A & G | 12 |
| 36 | UP! – Mood Tracker for Burnout and Bipolar | Netural | M | G | 1 |
| 37 | WorryTree: Anxiety Relief & CBT Diary | WorryTree Ltd | M | A & G | 4 |
| 37 | My Oasis: Anxiety Relief Game (also called My Oasis: Calming, Relaxing & Anxiety Relief Game) | Buff Studio | P | A & G | 1 |
| 37 | Heat Pad – Relaxing Surface | Padadaz | P | A | 1 |
| 38 | Happify | Happify, Inc. | P | A & G | 12 |
| 39 | Mine'd – Self Help Motivation | Mine’d Co. | P | A & G | 4 |
| 40 | Joyster: Self-Care, Mental Health Diary (Joyster: Daily Symptom Tracker) | Maria Paula Barcante | P | A & G | 3 |
|  |  |  |  | *Mean quality across all apps:* | 3.02  (*SD* = 3.27) |
